# Frequency of methicillin-resistant Staphylococcus aureus nasal colonization among patients suffering from methicillin resistant Staphylococcus aureus bacteraemia 

**DOI:** 10.12669/pjms.296.3911

**Published:** 2013

**Authors:** Nadia Aslam, Mateen Izhar, Naima Mehdi

**Affiliations:** 1 Nadia Aslam, M. Phil, Department of Pathology, Sargodha Medical College, Sargodha, Pakistan.; 2 Mateen Izhar, PhD, Head of Microbiology Department, Shaikh Zayed Hospital, Lahore, Pakistan.; 3 Naima Mehdi, M.Phil, Department of Microbiology, The Children Hospital and Institute of Child Health, Lahore, Pakistan.

**Keywords:** Bacteraemia, Nasal colonization

## Abstract

***Objective:*** To determine rate of nasal colonization in Patients suffering from bacteraemia caused by methicillin resistant Staphylococcus aureus.

***Methods:*** This descriptive cross sectional study was carried out in a tertiary ca re, University Teaching Hospital (Shaikh Zayed Hospital, Lahore) from October 2010 to August 2011. Nasal swabs were taken from patients suffering from MRSA bacteraemia and were plated on mannitol salt agar plates to isolate Staphylococcus aureus (S. aureus) which were then tested for oxacillin susceptibility.

***Results:*** Nasal colonization was present in 52.5% of patients suffering from MRSA bacteraemia.

***Conclusion:*** Nasal colonization rates with MRSA were high among patients suffering from MRSA bacteraemia especially in those undergoing dialysis or surgical procedures. Therefore, screening and nasal decolonization should be practiced in hospitals.

## INTRODUCTION

Staphylococcus. aureus is an important human pathogen and causes a wide range of suppurative infections.^1^^,^^2^ The presence of a microbe in or on a host with growth and multiplication of that micro-organism but without tissue damage, is termed colonization.^3^ S. aureus can colonize the anterior nares, and 25 to 30 percent of the population is colonized at a given time.^4^ Extranasal sites that can be colonized include skin, pharynx, perineum, gastrointestinal tract, vagina and axilla.^5^ Carriers of S. aureus are also two to nine times more likely than non carriers to have surgical-site infections.^4^ Colonized patients are the chief source of S. aureus in hospitals, responsible for clinical infections and can spread it to other patients.^4^^,^^6^ S. aureus carriage rates vary among different ethnic groups.^5^

This study was carried out to determine rate of nasal colonization in patients suffering from MRSA bacteraemia in a tertiary care hospital in Pakistan.

## METHODS

This descriptive cross sectional study was carried out in a tertiary care, University Teaching Hospital (Shaikh Zayed Hospital, Lahore) from October 2010 to August 2011. A total number of 4058 blood cultures were received from different patients during study. Blood cultures were first inoculated in tryptic soy broth for 48 hours and then on solid media (chocolate and MacConkey agar). The identification of S. aureus was made on colonial morphology, gram stain, catalase test, and deoxyribonuclease (DNase) test and/or coagulase test.^7^ For oxacillin susceptibility testing the S. aureus strains were inoculated on Mueller-Hinton agar plates. After placing 1-ug oxacillin disk, the plates were incubated at 35 C and the results were read after a full 24 hours according to CLSI (2010) recommendations.^8^

Fifty one consecutive methicillin resistant Staphylococcus aureus strains were isolated. Seven patients expired before nasal swab screening. Therefore nasal swabs were taken from 44 patients. Nasal swabs from the patients with MRSA bacteraemia were taken after taking written informed consent. To screen patients for S. aureus carriage, a dry, sterile swab was rotated four times in anterior 1.5cm of each nostril. Swab was plated on mannitol salt agar. After overnight incubation at 35°C, mannitol fermenting colonies were selected and sub cultured on a Blood agar plates (BAP; Oxoid).^9^ After overnight incubation, the colonies were screened by using DNase test and tube coagulase tests.^9^ S. aureus isolates were tested for oxacillin resistance by using 1-µg oxacillin disk as per recommendations of the Clinical Laboratory Standards Institute (CLSI).^8^ Percentage of patients having nasal colonization with MRSA was calculated.

## RESULTS

Nasal colonization was present in 23 patients out of 44 patients. (52.5% of patients) Distribution of nasal colonization among patients who were having different clinical conditions as source of bacteraemia is shown in Table-I. Out of 23 patients having nasal colonization, 15 patients were male and 8 patients were female. 

**Table-I:** Percentage of Nasal colonization among Patients suffering from MRSA blood stream infection according to Source of bacteraemia

Source of bacteraemia*	*Number of patients*	*Nasal colonization*	*Percentage*
CVP Line	5	3	60
Dialysis Line	6	6	100
Peripheral IV Line	8	1	12.50
Ventilator associated pneumonia	4	1	25
Pneumonia	10	5	50
Urinary tract infection	4	3	75
Surgical site infection	3	3	100
Skin and soft tissue infection	2	1	50
Unknown	2	0	0

*Clinical suspected or culture proven source of bacteraemia

## DISCUSSION

Preventing bloodstream infections with MRSA is important since infections caused by this antimicrobial-resistant pathogen are associated with considerable morbidity and mortality, and excess hospital costs. Colonization and infection by S. aureus are known to be significantly associated with infection among hospitalized patients.^6^ In most instances the development of MRSA bacteraemia is probably a two-stage process with acquisition of the organism and colonization of skin or superficial sites followed, after a variable period, by invasion of the bloodstream.^10^ Higher colonization rates are found in patients undergoing dialysis,^11^ higher nasal colonization rate was recorded in patients from dialysis unit by this study. Halablab MA and colleagues found increased rate of nasal colonization in males.^12^

Similarly, increased rate of nasal colonization in males was recorded during this study. MRSA colonization of nares, either present at admission to the hospital or acquired during hospitalization, increases the risk for MRSA infection.^13^ Rate of MRSA blood stream infection was found to be higher in males (64.7%) as compared to females (35.3%). Some other studies also depict the increased rate of MRSA infections in males.^12^^,^^14^^-^^16^ Most of the nosocomial S. aureus infections are caused by the patient’s own S. aureus cells already present on the skin or mucosal membranes before hospital admission in at least 80% of the cases. It can be said that more infections are of endogenous origin.^5^ Hence, increased rate of nasal carriage can predispose these patients to MRSA infections. Nasal colonization was recorded in 52.3% of patients included in the study, which is higher than in normal population (20-30%).^5^

Another study by Moniri R found similar rate of nasal carriage in hospitalized patients (52.6).^17^ Nasal carriage rates were found high especially in patients undergoing dialysis and in patients who had post-operative complications. A study by Donker and colleagues, found that nasal colonizers with MRSA are at 10 fold increased risk of surgical site infection with MRSA.^18^ Nasal carriage of MRSA among hospitalized patients can predispose patient to recurrent infections as well as MRSA can be transmitted to others.^17^ Decolonization of nasal and extra-nasal sites on hospital admission may reduce this risk.^19^^,^^20^

## CONCLUSION

In conclusion, frequency of nasal colonization with MRSA was found to be high among patients suffering from MRSA bacteraemia. MRSA nasal colonization can predispose the patients to increased risk of invasive infections or MRSA bacteraemia. Therefore, screening of patients for nasal colonization and eradication of MRSA from patients who are colonized, especially those undergoing dialysis and operative procedures, should be considered routinely to reduce incidence of MRSA infections in hospitals.
